# Engineering antimicrobial coating of archaeal poly-γ-glutamate-based materials using non-covalent crosslinkages

**DOI:** 10.1038/s41598-018-23017-x

**Published:** 2018-03-15

**Authors:** Makoto Ashiuchi, Yuichi Hakumai, Sawami Nakayama, Haruna Higashiuchi, Kosuke Shimada

**Affiliations:** 1 Department of Agriculture, Faculty of Agriculture, Kochi University, Nankoku, Kochi, 783-8502 Japan; 2 Course of Applied Bioresource Science, United Graduate School of Agricultural Sciences, Ehime University, Matsuyama, Ehime 790-8566 Japan

## Abstract

We are now entering a new age of intelligent material development using fine, sustainable polymers from extremophiles. Herein we present an innovative (but simple) means of transforming archaeal poly-γ-glutamate (PGA) into extremely durable polyionic complexes with potent antimicrobial performance. This new *supra*-polymer material (called PGA/DEQ) was subjected to nuclear magnetic resonance and X-ray diffraction spectroscopies to characterize in structural chemistry. Calorimetric measurements revealed its peculiar thermal properties; to the best of our knowledge, it is one of the most heat-resistant biopolymer-based polyionic complexes developed to date. PGA/DEQ is particularly useful in applications where surface functionalization is important, *e.g*., antimicrobial coatings. The spontaneously assembled PGA/DEQ coatings (without any additional treatments) were remarkably resistant to certain organic solvents (including chloroform), even at high salt concentrations (theoretically greater than those found in sea water), and various pH values. However, the pH-response tests also implied that the PGA/DEQ coatings could be removed only when concentrated citrate *di*-salts were used, whereas most crosslinked polymer composites (*e.g*., thermoset matrices) are difficult to recycle and treat downstream. We also discuss PGA/DEQ-immobilized surfaces that exhibit enigmatic microbicidal mechanisms.

## Introduction

Demand for biocidal polymers as coating materials that provide protection from the invasion of microbes is increasing. Surfaces contaminated with pathogenic organisms are vectors for the spread of pathogens, and affect those most vulnerable to infection. Quaternary ammonium (QA) compounds are used in a variety of commercial applications ranging from cosmetic preservatives to hospital disinfectants and sanitizers. They have many advantages over other biocides (*e.g*., phenols and aldehydes), including broad-spectrum antimicrobial activity, effectiveness over a wide pH range, low human toxicity, and lack of a detectable odour^1^. However, QA-based antimicrobial coatings are unsustainable owing to their amphiphilic properties, which presumably accelerate their liberation from surfaces and their immediate dispersion into various organic solvents and water. Moreover, they are small molecules (<1000 Da). The development of hierarchical assemblies of QAs with chemical durability (*e.g*., water-resistance) is therefore a promising yet challenging field of material nanotechnology.

Ashiuchi *et al*.^2^ first developed a stoichiometric ion complex comprising equal proportions of carboxyl groups from archaeal poly-γ-l-glutamate (l-PGA) and hexadecylpyridinium cations (HDP^+^) derived from QA compounds used in toothpaste. They called this self-assembling material l-PGA ion complex (PGAIC). PGAIC has a broad spectrum of antimicrobial activity (against food-poisoning bacteria, a prevalent species of *Candida*, filamentous fungi, and *Influenza* viruses)^2,3^, and has potential as a bio-based plastic. This new material could be useful in food-related engineering and pharmaceutics. However, the solvation assays developed for PGAICs^2,3^ also highlight the importance of improving their durability, in particular, moderate resistance against organic solvents including alcohols (*e.g*., ethanol) and chloroform. We herein present a new strategy to introduce multiple non-covalent crosslinkages into the essential architecture of PGAIC to develop engineered materials with excellent chemical durability. A sustainable PGAIC material was actually synthesized using a *bis*-QA (quinolinium) compound used in medicinal lozenges, called dequalinium cations (DEQ^2+^). In this article, the potential of DEQ-bound PGAIC (PGA/DEQ) materials as durable antimicrobial coatings is compared with that of traditional HDP-bound PGAIC (PGA/HDP) materials.

## Results

### Development of PGA/DEQ, a novel PGAIC material with non-covalent crosslinkages

**Figure 1:**
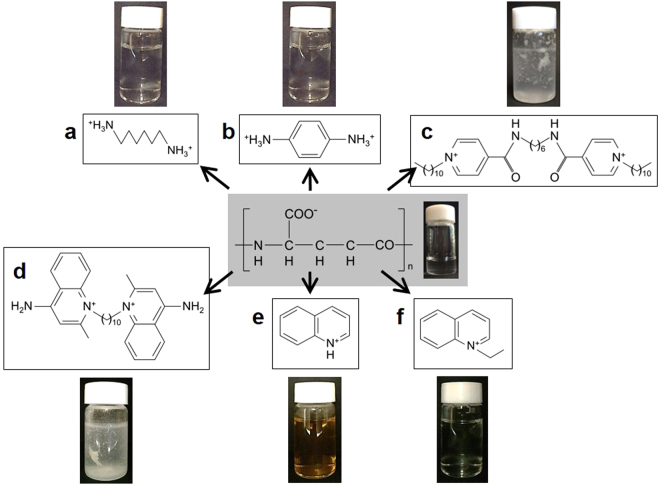
Development of poly-γ-L-glutamate (PGA) ion complexes (PGAICs) with multiple, non-covalent crosslinkages. In the reaction mixtures, the initial concentration (mg/mL) of PGA was 4.0 ± 0.2, indicating the presence of the anionic (carboxy) groups at ~31 mM, and the supply of crosslinker candidates (**a**), 1,6-diaminohexane; (**b**) *p*-phenylene diamine; (**c**) BDP^2+^; (**d**) DEQ^2+^; (**e**) quinolinium dichromate; and (**f**) quinoline ethiodide) was controlled so that the concentrations (mM) of their cationic (amine) groups reached the same levels as the anionic groups. The increases in turbidity virtually implied the accumulation of water-insoluble PGAICs.

In the present study, several crosslinker candidates were used to synthesize PGAICs (Fig. 1). Diamine-type compounds (*e.g*., hexamethylene (*viz*., aliphatic; panel a) and phenylene (*viz*., aromatic; b) diamines) did not meet the requirements for PGAIC materials, whereas *N*, *N’*-hexamethylenebis (4-carbamoyl-1-decylpyridinium) (BDP^2+^, a *bis*-pyridinium compound) was applicable (c), as was HDP^+^ (a *mono*-pyridinium compound)^2,3^. DEQ^2+^ (a *bis*-quinolinium compound) was useful as well (d), but *mono*-quinolinium compounds (e and f) were not preferred for the formation of water-resistant complexes. This implies unexpected performance (*e.g*., extreme solvent resistance and sustainable functionality) in PGA/DEQ as a novel PGAIC product with multiple, non-covalent crosslinkages. The “soakage” tests for PGA/DEQ actually indicated a dramatic improvement in the resistance of PGAIC materials to all the organic solvents tested (Table 1). In contrast, another new product called PGA/BDP has yet to overcome the concerns about chemical stability that apply to traditional PGAIC products (*e.g*., PGA/HDP), suggesting that quinolinium-based crosslinkers are more beneficial than pyridinium-based crosslinkers in developing durable PGAIC materials.

**Table 1:** Comparison of the solvation properties of *bis*-quinolinium- and *mono*- and *bis*-pyridinium-bound materials of PGAIC.

**Samples** ^**a**^	**Organic solvents** ^**b**^						
	MeOH	EtOH	CHCl_3_	DMSO	NMPy	Acetone	Hexane
PGA/DEQ	−^**c**^	−	−	−	−	−	−
DEQ^2+^	+^**c**^	±^**c**^	+	−	−	−	−
PGA/HDP	+	+	+	−	−	−	−
HDP^+^	+	+	+	+	−	−	−
PGA/BDP	+	+	+	−	+	−	−
BDP^2+^	+	+	+	+	+	−	−

^a^PGAICs and their cationic partner chemicals were added at the theoretical concentration of 1 wt%. ^**b**^Abbreviations: MeOH, methanol; EtOH, ethanol; CHCl_3_, chloroform; DMSO, dimethyl sulfoxide; NMPy, *N*-methyl-2-pyrolidone. ^**c**^Symbols: +, soluble; −, insoluble; ±, almost insoluble. Essentially, natural PGA (PGA^*n−*^; namely poly-γ-glutamate) is insoluble in any organic solvents, whereas only DMSO can solubilize free PGA (PGAH_*n*_; called poly-γ-glutamic acid), which is artificially protonated^2^.

### Structural features of PGA/DEQ

**Figure 2:**
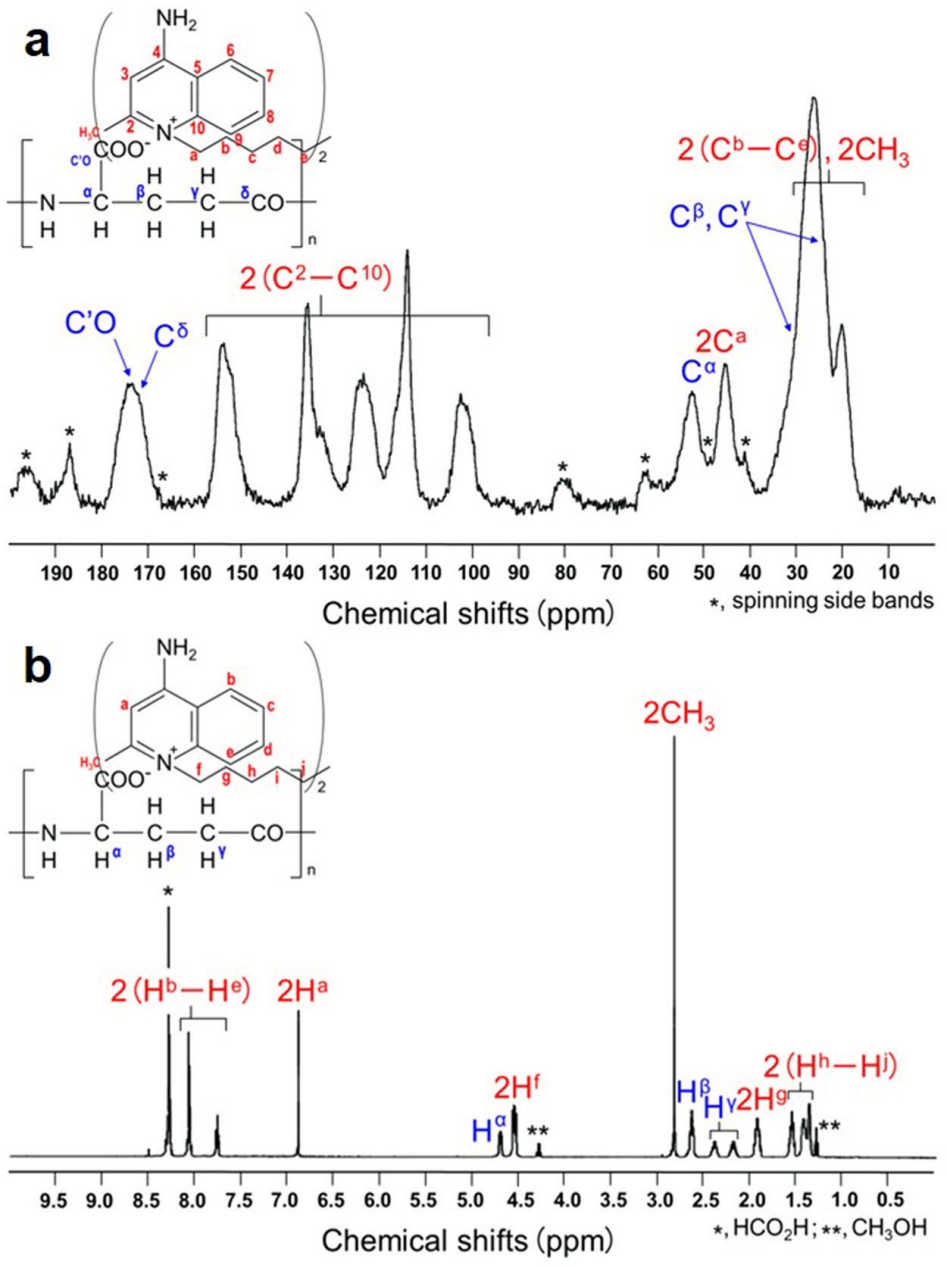
(**a**) ^13^C NMR and (**b**) ^1^H NMR spectra of PGA/DEQ; the insets illustrate assigned PGA ion complex (PGAIC) structures.

We will now describe the effective synthesis and nuclear magnetic resonance (NMR) analysis of PGA/DEQ. After its synthesis, little L-PGA (<10 wt% of the initial amounts; *n* = 5) was detected in the aqueous phase of the reaction mixtures using published procedures^4^, confirming that L-PGA was almost completely converted to a water-insoluble form of PGAIC by interacting with DEQ^2+^. By ^13^C cross polarization and magic angle spinning (^13^C CP-MAS) NMR spectroscopy method, we successfully characterized the chemical shifts that correspond to L-PGA and DEQ^2+^ from the spectrum of PGA/DEQ (Fig. 2, panel a), while in particular the corresponding peaks to DEQ^2+^ were much broader than those of DEQ^2+^ itself (Supplementary Fig. 1a), suggesting development in solid-state PGA/DEQ of the molecular interaction between DEQ^2+^ (as smaller) and L-PGA (as higher moieties). Next, the proton signals found in b were assigned as follows: *bis*-quinolinium rings (chemical shifts 2 H^a^ and 2(H^b^–H^e^), 6.88 and 8.30–7.78; relative intensities, 1.00 and total 3.18), αCH-PGA (H^α^, 4.72; 0.92), *bis*-quinolinyl CH_2_ (2 H^f^, 4.55; 2.10), *bis*-quinolinyl CH_3_ (2CH_3_, 2.83; 3.17), βCH_2_-PGA (H^β^, 2.63; 1.82), γCH_2_-PGA (H^γ^, 2.40–2.20; total 1.84), CH_2_-DEQ^2+^ (2 H^g^, 1.95; 2.05), and (CH_2_)_3_-DEQ^2+^ (2(H^h^–H^j^), 1.57–1.38; total 6.18). Hence, PGA/DEQ was determined to be a stoichiometric ion complex comprising equal proportions of the carboxyl (*viz*., negatively-charged) groups of PGA and the quinolinium (*viz*., positively-charged) moieties of DEQ^2+^, which also demonstrates the feasibility of DEQ^2+^ as a potent crosslinking reagent for synthesizing durable PGAICs.

### Thermal characteristics of PGA/DEQ

**Figure 3:**
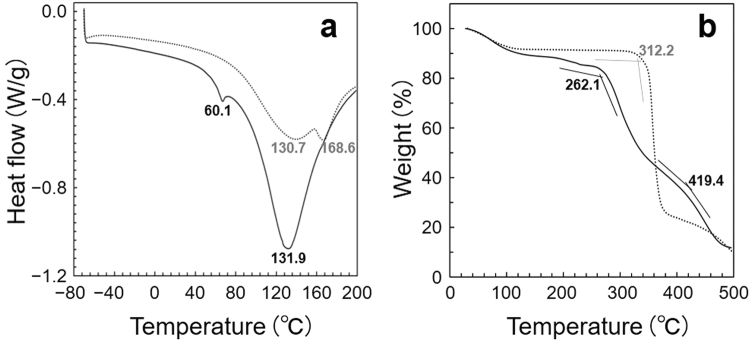
Calorimetric investigations of PGA/DEQ. Panels (**a**) differential scanning calorimetry (DSC) and (**b**) thermogravimetry (TG) profiles of PGA/DEQ (─) and authentic DEQ^2+^ (……).

The calorimetric measurements of PGA/DEQ (Fig. 3) revealed that the minor and major melting points (*Tm*s) were 60.1 °C (transition enthalpy, *ΔH* = 1.896 J/g) and 131.9 °C (219.1 J/g), respectively (panel a, *solid line*). PGA/DEQ, virtually a cross-linked polymer product, can retain its potential as a thermoplastic. We also found that the major and minor *Tm*s of DEQ^2+^ were 130.7 °C (*ΔH* = 114.3 J/g) and 168.6 °C (8.679 J/g), respectively (a, *dotted line*). Interestingly, the *ΔH* value of PGA/DEQ (weight ratio, 1: 4) was nearly twice that of authentic DEQ^2+^, and was comparable with that of 100% crystalline chemically-synthesized nylons (*e.g*., 241 J/g of α-phase and 239 J/g of γ-phase)^5^. The *Tm* of PGA/HDP, however, was only ~60 °C (*ΔH* = 74.08 J/g)^2^: close to that of the former (or minor) *Tm* of PGA/DEQ. This may suggest the ubiquitous thermal manner in archaeal L-PGA-based PGAICs, while it seems likely that the former (or major) *Tm* of DEQ^2+^ mainly involves the thermal plasticity of PGA/DEQ. A recent article described the comparatively high *Tm* (157 °C) yet small *ΔH* (2.3 J/g) values of a poly-ε-L-lysine ion-complex^6^. PGA/DEQ is, to the best of our knowledge, one of the most heat-resistant bio-based chiral nylon (or *iso*peptide)^7^ ion complex materials developed to date. Figure 3 also showed that the decomposition (*Td*) of PGA/DEQ started at 262.1 °C (panel b). In contrast to DEQ^2+^ (*dotted line*), the weight of PGA/DEQ decreased in two steps during the rise in temperature (*solid line*), implying that the configuration of theoretical PGA/DEQ oligomers (or digests) accumulated in the late stage of decomposition resulted in a superior heat resistance to that of DEQ^2+^ itself.

### Characterization of *micro*structures in PGA/DEQ

**Figure 4:**
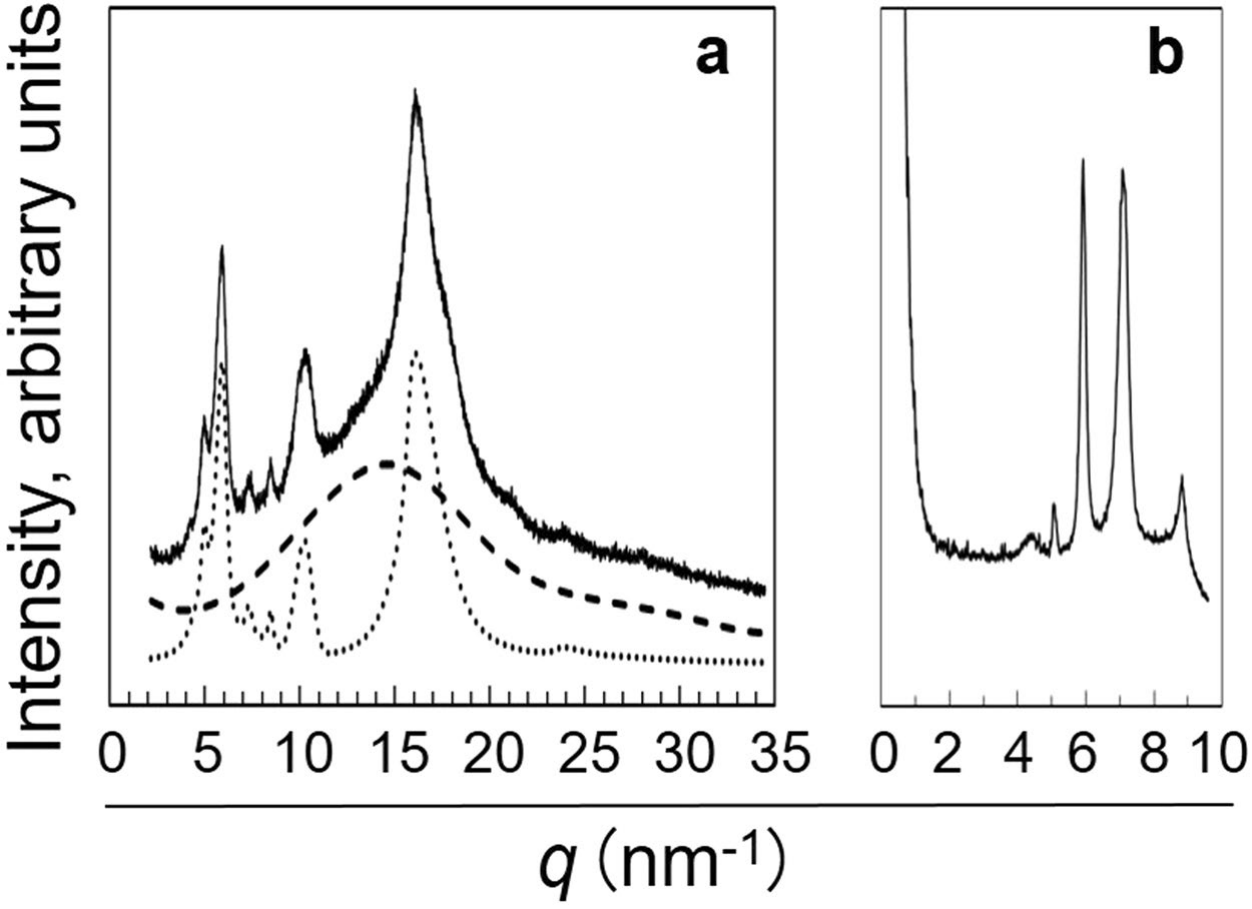
XRD analysis of PGA/DEQ. Panels (**a**) WAXD (, , crystalline diffractions; , amorphous scattering) and (**b**) SAXS diagrams.

The thermal properties of plastics essentially depend on the crystallinities of the materials, and the X-ray diffraction (XRD) analysis provides an insight into the structural features of the crystalline moieties of polymers. Figure 4 describes the wide-angle X-ray diffraction (WAXD) diagram of PGA/DEQ (panel a, *solid line*); a curve fitting model^8,9^ was further applied to separate the crystalline diffraction pattern (*dotted line*) from amorphous scattering (*broken line*). The crystallinity of PGA/DEQ was then calculated to be ~45% by dividing the integrated intensity of crystalline areas by the overall scattering intensity. It is also noteworthy that multiple (at least five) harmonic peaks were detected from 5 to 10 nm^−1^ of the *q* values in addition to the major crystalline peak at 16 nm^−1^. These extraordinary peaks indicate self-oriented assemblies of the crystalline moieties, specifically in the PGA/DEQ *micro*structure, because corresponding peaks were absent from the original *micro*structures of both PGA and DEQ^2+^ (Supplementary Fig. 2). Small-angle X-ray scattering (SAXS) analysis was performed to determine the evolved crystalline structures (Fig. 4, panel b). The profile of observed peaks revealed the presence of a peculiar harmonic, which does not correspond to any of the harmonics previously identified, including those of typical lamellar structures^10–12^. Hereafter, we will refer to the structures found in the PGA/DEQ *micro*structures as atypical crystalline assemblies.

### Performance of PGA/DEQ as antimicrobial coatings

**Figure 5:**
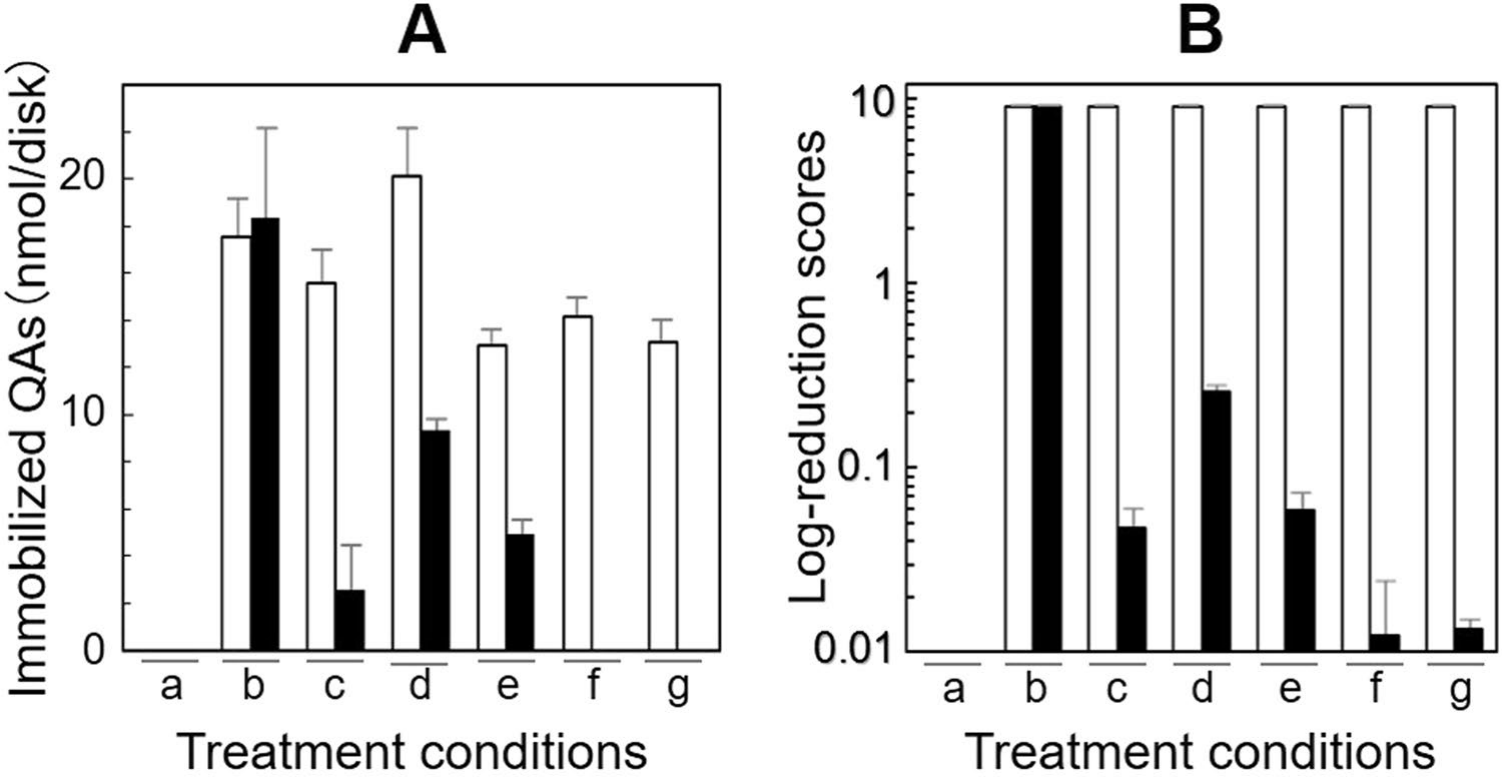
(**A**) Chemical durability and (**B**) functional sustainability of PGA/DEQ (▯) and PGA/HDP (▮) coatings. The basal (a) or a PGAIC-coated (b) disk was soaked into 2 mL of the indicated solvents (c, EtOH; d, CHCl_3_; e, 1.5%; f, 3.0%; and g, 5.0% NaCl) and incubated at room temperature while gently shaking for 30 min. This “soaking” process was repeated ten times per disk, though almost of the PGA/HDP coatings are usually removed after the first “soaking”, in particular using (c) or (g). The resulting disks were washed three times each with 2 mL of distilled water and finally dried at 60 °C for 1 h. The immobilized QAs and log-reduction scores (*n* = 15 each) were determined, based on the spectroscopic characteristics of BPBICs formed *onsite* (*see* also Supplementary Figs 10–12) and the enumeration of living microbial cells, respectively (*see* the Method section).

As shown in Fig. 5–panel A, PGA/HDP coatings (*black bars*; columns b) on the plastic surfaces (a) were stable during water-soaking; however, they were readily removed by the indicated organic solvents (c and d) and concentrated salt solutions (e–g). In contrast, the PGA/DEQ coatings (*white bars*) on the same materials (a) had excellent durability against all the solvents (or chemicals) tested (b–g). The morphology of PGAIC-coated *micro*fibers on these plastic surfaces was further observed using a scanning electron microscope (SEM) (Supplementary Fig. 3). The transformation of PGAIC into durable structures led to the further sustainable functionality of the antimicrobial coatings. The antimicrobial performance of the PGA/HDP coatings (Fig. 5–panel B, *black bars*) disappeared after soaking in the indicated solvents (c–g) other than water (b), whereas a PGA/DEQ-coated disk (Fig. 5B) resulted in the dramatic suppression of microbial proliferation (*white bars*) under any treatment conditions (b–g; Supplementary Fig. 4). Compared with the normal proliferation of *Escherichia coli* (~1.3 × 10^9^ colony-forming units (CFU)), the log-reduction scores of PGA/HDP and PGA/DEQ coatings (after treatment) were estimated to be almost zero and approximately nine, respectively. These PGAIC coatings could, therefore, contribute to the *micro*structure-based polymer engineering of antimicrobial (bioactive) coatings.

### Controllable removability of PGA/DEQ coatings

**Figure 6:**
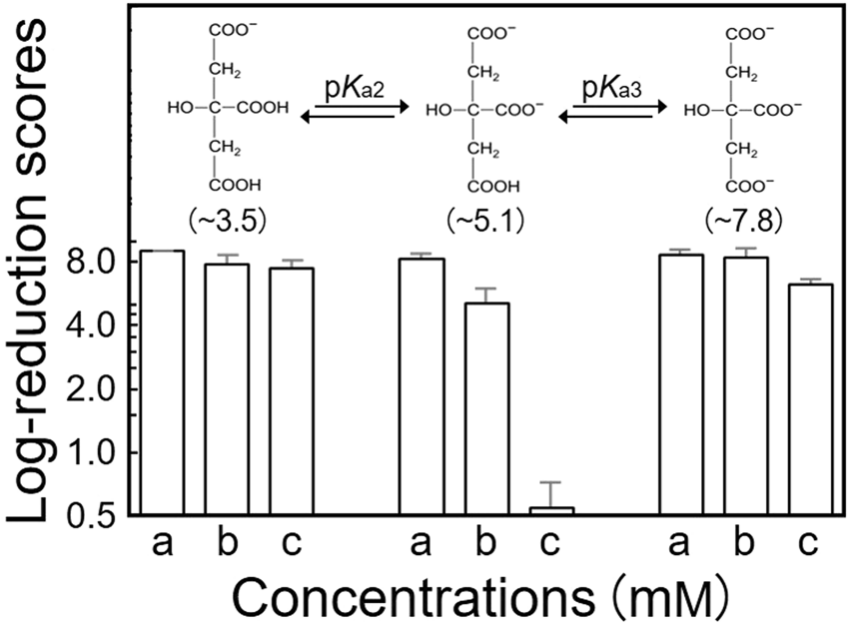
Controlled removal of PGA/DEQ coatings using electrochemically different forms of citrate. The “soaking” processes and the determination of log-reduction scores (*n* = 3) were essentially conducted according to the investigations described in Fig. 5. The insets illustrate the predicted structure of the citrate salts used, and the approximate pH values of their solutions can be found in parentheses below. Concentrations (a), 5; (b), 50; and (c), 100 mM.

Further investigation on the sustainability of PGA/DEQ as antimicrobial coatings (*viz*., the pH-response tests) accidentally provided a possible strategy for the controlled removal (or enforced termination) of PGA/DEQ from the modified surfaces. Our data (Supplementary Fig. 5) implied the significance (or specificity) of citrate *di*-salts (*theoretical* p*K*a2 = 4.75). In fact, highly concentrated citrate *di*-salts were the best candidate mediators of PGA/DEQ removal (Fig. 6), whereas other electrochemical forms, *e.g*. citrate *mono*- (p*K*a1 = 3.09) and *tri*-salts (p*K*a3 = 6.41), were essentially ineffective. Furthermore, we used quantitative assays to verify that the PGA/DEQ molecules were exclusively eliminated following soaking in the concentrated *di*-salts (*see* the Methods section). Crosslinked polymer composites (*e.g*., thermoset matrices) are usually difficult to recycle^13,14^. Further, the strategies for their downstream (waste) treatment are also limited. Finding a means of controllably removing (and possibly recycling) PGA/DEQ will help to enhance its value as a functional supramolecular polymer^15^ for versatile prophylactic coatings (or plastics/fibres)^2,3,16^.

### PGAIC-immobilized surfaces carrying potent microbicidal mechanism

A poly-ε-L-lysine ion complex had a dramatically reduced antimicrobial activity compared with native poly-ε-L-lysine^6^, but we found that the PGAIC solutions were quite similar to solutions of the corresponding partner surfactants in an assessment of minimal inhibition concentrations (MICs)^3^. In the present study, the average amount of PGA/DEQ immobilized on the surfaces of a basal disk was determined to be 17.6 μg; if the PGA/DEQ coatings were all released in 5 mL of the culture medium, the final concentration had the potential to reach 3.52 ppm. Because the concentration of the liberated DEQ^2+^ moieties was ~22.2 nmol (*via* complete decomposition of the coatings), the maximal concentration was theoretically calculated to be 4.44 μM (or 2.35 ppm). Possibly, there is a microbicidal factor that operates at significantly lower doses than the MIC values for *E. coli* cells in the original (free) DEQ solutions (Supplementary Table 1), as we have observed the potent, sustainable microbicidal performance of PGA/DEQ-coated surfaces (Supplementary Figs 6 and 7). In the next stage of PGAIC research, it may be interesting to elucidate such peculiar and potent molecular strategies by investigating them on solid interfaces (Supplementary Fig. 8).

## Discussion

Using a fine (sustainable) polymer from extremophiles, we have succeeded in developing a novel antimicrobial coating, showing the antithetical qualities of extreme durability and briefly controllable removability. This archaeal polymer, *namely* stereoregular PGA, possesses a peculiar functionality that enables it to bind cooperatively to various cationic compounds such as some critical metals^17^ and organic surfactants (Supplementary Fig. 9).

PGAICs can be transformed into versatile nanofiberplastics^2^ and safe dispersants to create antimicrobial surfaces, and they may be used to contribute to hygienic control and infection prophylaxis in various public facilities such as schools, hospitals, hotels, and transportation^3^, resulting in a decreased risk of airborne infections, contagions, and serious pneumonia, which are often fatal. Engineering advanced functional (*e.g*., controlled capture–killing) coatings would eventually prevent unforeseen epidemics caused by new contagions.

## Methods

### Materials

Archaeal PGA was isolated from a *Natrialba aegyptiaca* culture medium^2,3^. Two healthcare compounds (or cationic surfactants) suitable for PGAIC synthesis, *i.e*., HDP^+^ and DEQ^2+^, were purchased from the Tokyo Chemical Industry (Japan) and Sigma Co. (St. Louis, MO, USA), respectively., Salts of BDP^2+^ were obtained from Wako Pure Chemicals (Osaka, Japan) because it is a commercially available *bis*-QA (pyridinium) compound^18^. All other chemicals were of analytical grade.

### PGAIC synthesis

PGA/DEQ was synthesized by mixing a 2 wt% L-PGA solution with four times the volume of 1 wt % DEQ^2+^ solution (corresponding to approximately 48 mol% of the carboxyl groups contributed by the PGA molecules). Pellets were collected by centrifugation at 12000 *g* for 10 min, then washed several times with distilled water at 80 °C, soaked in pure methanol (>99.8 wt% MeOH) for 1 h at room temperature to remove the accidentally liberated DEQ^2+^, and lyophilized. The other (traditional) products of PGAIC (*e.g*., PGA/HDP) were prepared according to published procedures^2,3^.

### NMR spectroscopy

#### Solid-state analysis

^13^C CP-MAS NMR spectroscopy was performed on the lyophilized samples (50 mg each) using a Bruker DSX300 spectrometer (La Jolla, CA, USA) operated at 75.5 MHz. We scanned 40000 and 28583 times at 5.5 kHz for DEQ^2+^ and PGA/DEQ, respectively.

#### Liquid-state analysis

A lyophilized PGAIC sample (10 mg) was dissolved in 1 mL of *di*-deuterated formic acid (DCO_2_D), and analysed using a Bruker AVANCE500 spectrometer operated at 500 MHz. The NMR signals were assigned based on the chemical shifts (ppm), their relative intensities, and coupling patterns, and further comparisons with the signals of indispensable (or starting) materials: L-PGA (*refer to* ref.^2^) and DEQ^2+^ (*see* Supplementary Fig. 1).

### Calorimetric measurements

DSC and TG analysis of lyophilized PGA/DEQ (~5 mg) were performed at a heating rate of 10 °C/min under a nitrogen atmosphere using Seiko DSC EXTRA6000 and SSC5200 equipment (Chiba, Japan), respectively.

### XRD analysis

WAXD and SAXS measurements were used to decipher the short-range order and the large-scale structure of the crystalline regions of PGA/DEQ, respectively. WAXD was carried out on a Rigaku SmartLab system (Tokyo, Japan). Because the incident X-ray beam from a line focus tube (Cu, operating at 40 kV and 30 mA) is generally divergent, it was transformed into an intense parallel Kβ radiation-free beam using a Göbel mirror. This beam (from an incident 5° Soller slit against a 10-mm longitudinal limits slit) was achieved using an equatorial axial 0.114° Soller slit in the secondary beam path, and the scattering angle 2*θ* varied from 3° to 90° in reflection (*θ*–2*θ* configuration) with a step of 0.01° and a speed of 5°/min. For SAXS, we operated a Rigaku NANO-Viewer IP instrument at 40 kV and 30 mA, the flight path and the sample chamber of which were under *vacuum*. The Cu Kα radiation (from three pinhole slits) had a wavelength (λ) of 1.54 Å, and the sample-to-detector distance was estimated to be 368 mm when silver behenate was used as a distance calibration standard. The scattered intensity *I* (*q*) was recorded in the range 0.08 < *q* < 8.0 nm^−1^, where *q* is the scattering vector defined as *q* = (4π/*λ*) sin (*θ*). During this XRD analysis, the PGA/DEQ sample was investigated without thermal treatment.

### Coating of PGA/DEQ on plastic surfaces

A recent study dealt with the performance of PGA/HDP as a widely applicable adhesive on the surfaces of versatile plastics (and other materials), in which additional chemical modifications (*e.g*., non-biodegradable crosslinking) and thermal treatments (*e.g*., heating) were omitted from the coating process^3^. Hereafter (in situations that require the further strengthening of PGAIC coatings)^2,3^, it is urgently necessary to demonstrate the potential of PGA/DEQ as an antimicrobial coating compared with commercially available (or ready-made) plastic materials (*e.g*., HIYEX non-woven cloths). The kinetics of PGAIC synthesis revealed the occurrence of potent cooperative interaction between PGA and the QA-type surfactants to form the stoichiometric ion complexes (Supplementary Fig. 9). In the present experiment, the direct formation of PGAIC (*viz*., PGA/DEQ and PGA/HDP) coatings on the material surfaces was performed using a modified layer-by-layer^19^ (or *onsite* coating) method (Supplementary Fig. 10A). First, we coated 40 μL of ~ 0.12 wt % L-PGA solution onto the surface of a disk (diameter 12 mm) of HIYEX HO-503M non-woven plastic cloth (or sheet) (Kuraray, Tokyo, Japan), and dried it at 28 °C until its weight had been reduced by almost half. The PGA-mounting disks were immersed briefly in warmed solutions of QA (1 mL/disk), *i.e*., DEQ^2+^ (1 wt%; 80 °C) and HDP^+^ (3 wt %; 60 °C), and dried at 60 °C for 1 h to introduce PGAIC assemblies *onsite*. Based on the differences in the solvation properties between the QAs and their corresponding PGAICs (Table 1), excess unbound QAs were thoroughly removed from the surfaces using methanol and dimethyl sulfoxide (DMSO), respectively (Supplementary Fig. 6A, step d). Because of the excellent water resistance of the PGAIC coatings^2,3,16^, the disks were soaked in distilled water five times (2 mL/disk) at 28 °C, dried again at 60 °C, and used as PGAIC-coated materials.

### Scanning Electron Microscopy

A Nihon Denshi Field Emission Scanning Electron Microscope JSM 6700 F (Tokyo, Japan) was operated with an acceleration voltage of 5 kV to produce the indicated SEM images.

### Investigation of surface-immobilized PGAIC coatings

Bromophenol blue (BPB; Nakarai, Kyoto, Japan) is a water-soluble (anionic) dye that enables the visualization of various quaternary ammonium compounds^20,21^. A BPB-staining method^3^ was modified to quantify QA moieties immobilized on the surfaces (Supplementary Fig. 10B). PGAIC-coated materials were immersed in 0.04 w/v % BPB solution (1 mL/disk) at 28 °C for 10 min (step e), washed five times with running water (5 mL/disk) to remove unbound BPB (step f), and dried at 60 °C. The materials were dyed blue because water-insoluble complexes comprising BPB anions and QA cations (*viz*., DEQ^2+^ and HDP^+^) were formed *onsite* (Supplementary Fig. 11). Hereafter, this blue compound will be referred to as BPB ion complex (BPBIC); it is readily soluble in methanol (Supplementary Fig. 10, step g), and the spectrophotometry of BPBIC can be used for the quantitative analysis of surface-immobilized PGAIC coatings.

### Spectrophotometric assay of BPBICs

Water-insoluble BPBICs were immediately formed in a mixture comprising BPB solution (0.04 wt%; at neutral pH) and a concentrated solution that contained virtually half-molar amounts of DEQ^2+^ or equimolar amounts of HDP^+^. Pellets were collected by centrifugation, and washed six times with water at 60 °C. The lyophilized BPBICs were then dissolved in methanol (>99.5 wt %), and the spectrophotometry of the resulting solution was conducted using a GE Healthcare GeneQuant 100 instrument (Tokyo, Japan) to obtain the absorption spectra of the DEQ-bound BPBIC (BPB/DEQ) and the HDP-bound BPBIC (BPB/HDP) (Supplementary Fig. 12, panel a). The calibration curves of BPB/DEQ and BPB/HDP were then constructed based on the absorbance at 590 nm as their common maximum absorption wavelength (b and c). Because the predicted molecular masses (Da) of BPB/DEQ and BPB/HDP were 1796.6 and 974.51, respectively, the molar extinction coefficients (M^−1^·cm^−1^) of BPB/DEQ and BPB/HDP were calculated to be 8.99 × 10^4^ and 3.44 × 10^4^, respectively.

### Assessment for antimicrobial performance

Although PGA is virtually non-toxic to humans and even pathogenic microbes, PGAICs exhibit a “broad-spectrum” antimicrobial activity with regard to *Influenza* viruses and fungi^2,3^, suggesting that the desirable performance of PGAIC is dependent on the potential of its corresponding QA moieties (*e.g*., HDP^+^ in PGA/HDP and DEQ^2+^ in PGA/DEQ). The antimicrobial spectrum of DEQ^2+^ is quite extensive (Supplementary Table 1), and is essentially the same as that of HDP^+ ^^2,3^. Herein, we focused on the observation that the anti-*E. coli* activities of PGAICs are often moderate compared with their other antimicrobial activities, because the efficacy of PGAIC-coated surfaces against *E. coli* cells is profoundly influenced by the stability (or durability) of PGAIC as a coating. It is worth noting that *E. coli* is an environmental (water) pollution indicator species^16^. In the present experiment, viable *E. coli* cells (~1.7 × 10^5^ CFU) were first inoculated into 5 mL of Luria–Bertani (LB) medium^22^, then a PGAIC-coated disk (*see* above) was introduced and the cells were cultured at 37 °C for 16 h. The culture suspension (0.1 mL) was spread onto a DAIGO agar (Nihon Pharmaceutical) plate and incubated at 37 °C for 12 h to determine the number of *E. coli* colonies, *viz*. the direct plating method for the enumeration of living cells^16,23,24^. When >300 colonies had formed on the plate, the samples were additionally diluted by 10 times and the enumeration test was repeated. The antimicrobial performance of the PGAIC coatings was assessed using log-reduction scores, which can be calculated using the following equation: log_10_ *x*/*y*, where *x* and *y* correspond to the counts of viable cells in the LB cultures harbouring the original (*thus* non-coated) and PGAIC-coated disks, respectively.

## Electronic supplementary material

Supplementary Information
