# Supplementary material for: Engineering antimicrobial coating of archaeal poly-γ-glutamate-based materials using non-covalent crosslinkages
Source: Sci Rep. 2018 Mar 15;8:4645. doi: 10.1038/s41598-018-23017-x (PMC5854621; doi:10.1038/s41598-018-23017-x)
Supplement: Supplementary file 1 — Supplementary Information [file 41598_2018_23017_MOESM1_ESM.pdf]

Supplementary Information

Engineering antimicrobial coating of archaeal poly-γ-glutamate-based materials using non-covalent crosslinkages

Makoto Ashiuchi[1,2,*], Yuichi Hakumai[2], Sawami Nakayama[1], Haruna Higashiuchi[1], Kosuke Shimada[1]

[1]*Department of Agriculture, Faculty of Agriculture, Kochi University, Nankoku, Kochi 783-8502, Japan.* [2]*Course of Applied Bioresource Science, United Graduate School of Agricultural Sciences, Ehime University, Matsuyama, Ehime 790-8566, Japan.*

*Correspondence and requests for materials should be addressed to M.A. (email: ashiuchi@kochi-u.ac.jp)

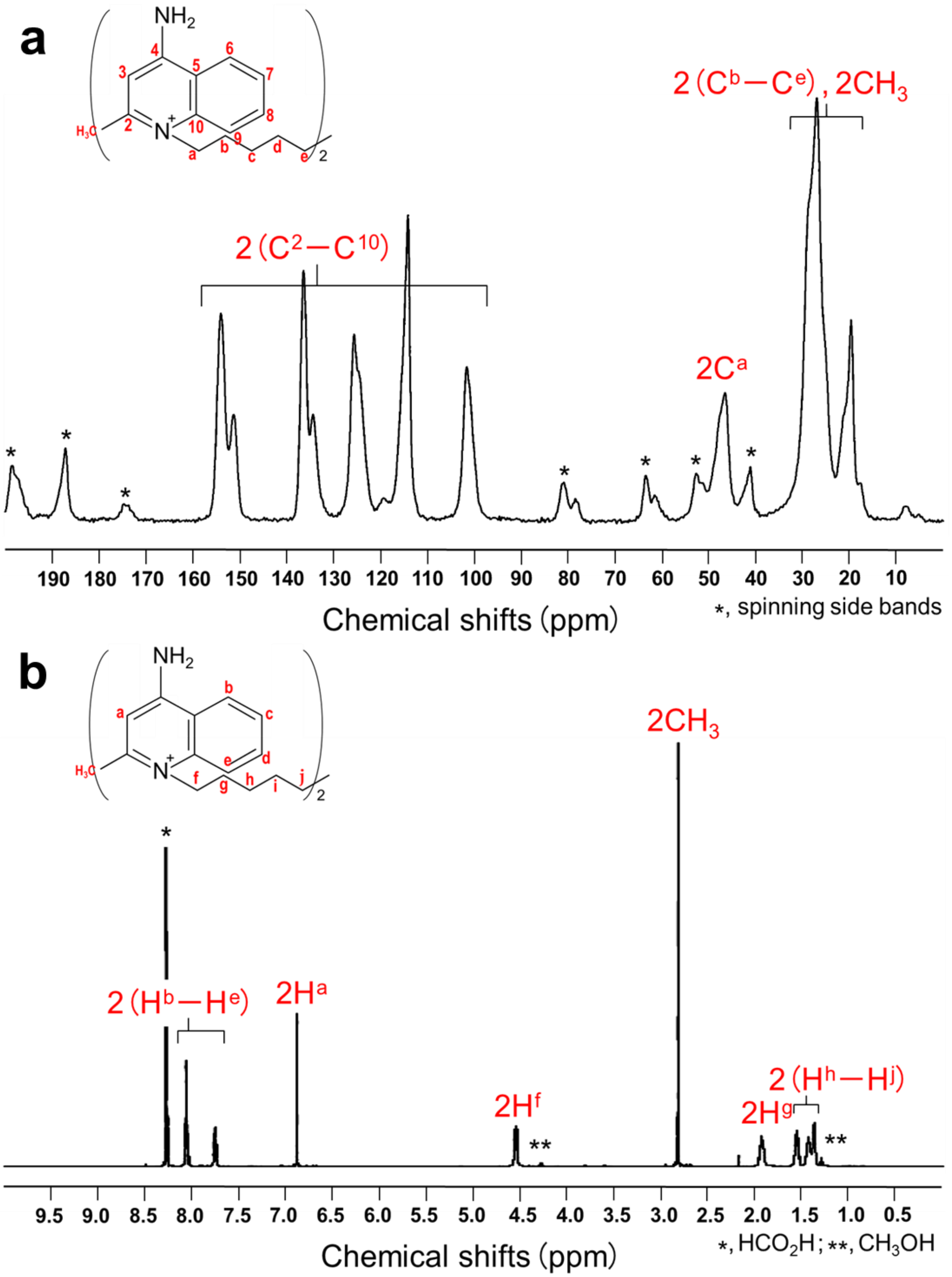

Supplementary Figure 1. (a) ^{13}C NMR and (b) ^{1}H NMR spectra of authentic DEQ^{2+}; the insets illustrate assigned DEQ^{2+} structures.

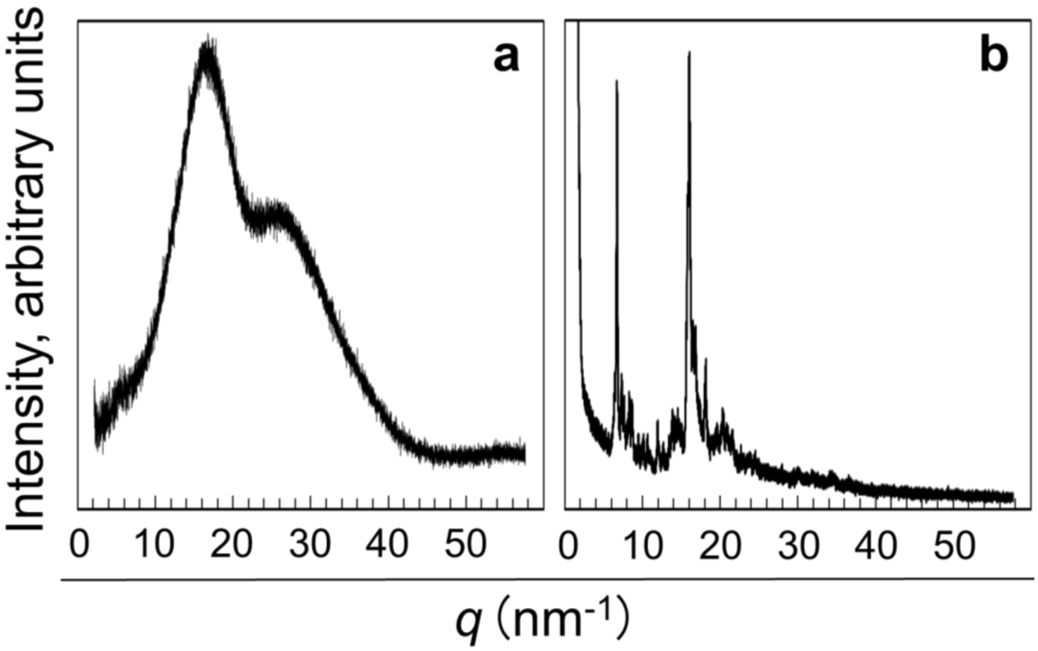

Supplementary Figure 2. WAXS spectra of PGAIC-precursor standards. Authentic samples (**a**), poly-γ-glutamate (PGA; from Wako, Japan); and (**b**) dequalinium *di*-chlorides (DEQ^{2+}; from Sigma Co., USA).

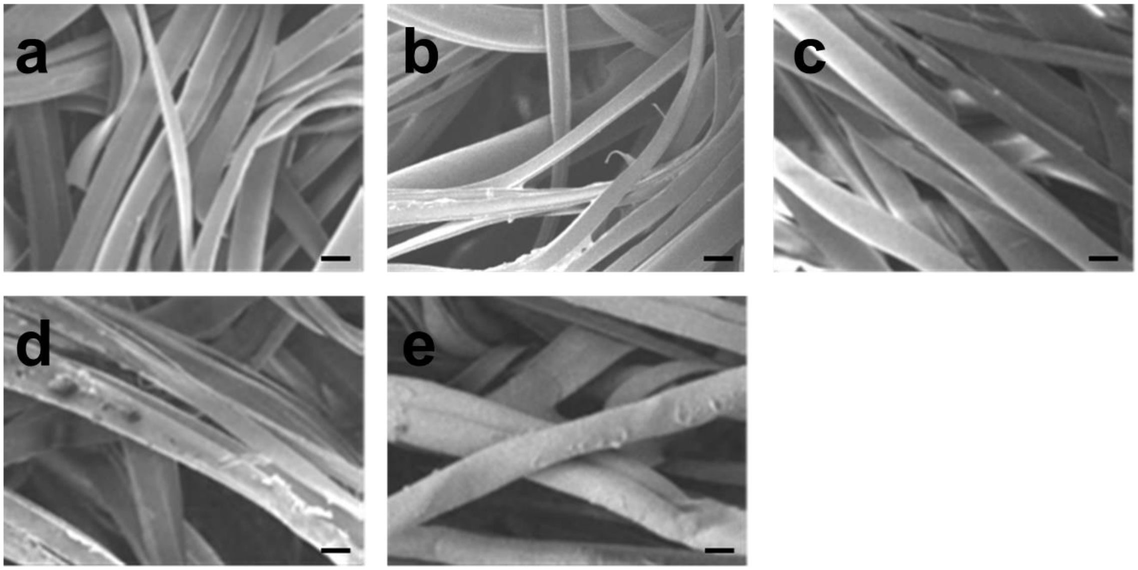

Supplementary Figure 3. Electron microscopy of PGAIC-coated *micro*fibers on plastic surfaces.

SEM images, from (**a**) a non-coated HIYEX non-woven plastic cloth (or sheet) (from Kuraray, Japan); the PGA/HDP-coated sheets (**b**) before and (**c**) after the EtOH (>99.5 wt%)-soaking process (*see* Fig. 5); and the PGA/DEQ-coated sheets (**d**) before and (**e**) after the same severe treatment. The length of the black bar is 10 μm. In particular, the image **e** indicates the excellent durability of the PGA/DEQ coatings against alcohols.

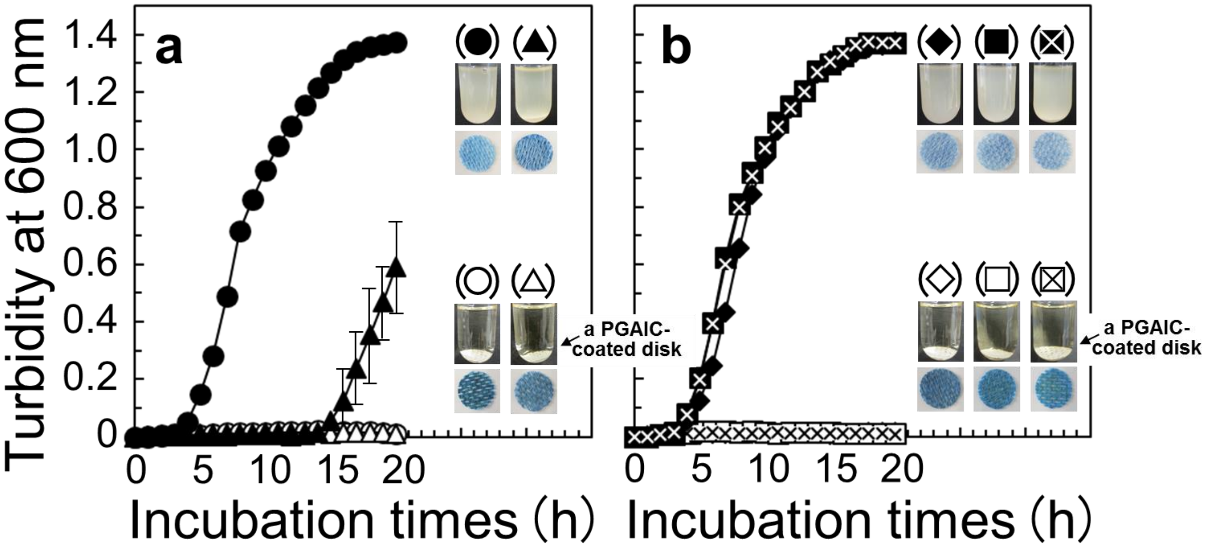

Supplementary Figure 4. Growth curves of *E. coli.*

The viable cells (~1.7×10^5 CFU) were first inoculated into Luria–Bertani (LB) media (5 mL), each carrying a disk (12 mm *dia.*) formed from PGA/DEQ-coated sheets (*open symbols*) and PGA/HDP-coated sheets (*closed symbols*) treated in the following ways: (**a**) soaking in EtOH (*circles*) or $CHCl_3$ (*triangles*); and (**b**) soaking in 1.5% NaCl (*diamonds*), 3.0% NaCl (*squares*), or 5.0% NaCl (*squares with crosses* (or *ballet boxes with an x*)). The (net) growth rates of the colonies were then estimated by monitoring the culture turbidity at 600 nm using a spectrophotometer ($n = 3$). The standard deviations observed in the latter treatment were actually $< 5\%$ (0 to a maximum of 0.04). Symbols in parentheses represent the images of the cultures (*top*) acquired at the end of 36-h cultivation; the BPB-stained disks (*bottom*) were essentially the same as the PGAIC-coated sheets used in the experiments, the darkness of which briefly corresponded to the quantity of PGAICs retained on the surfaces.

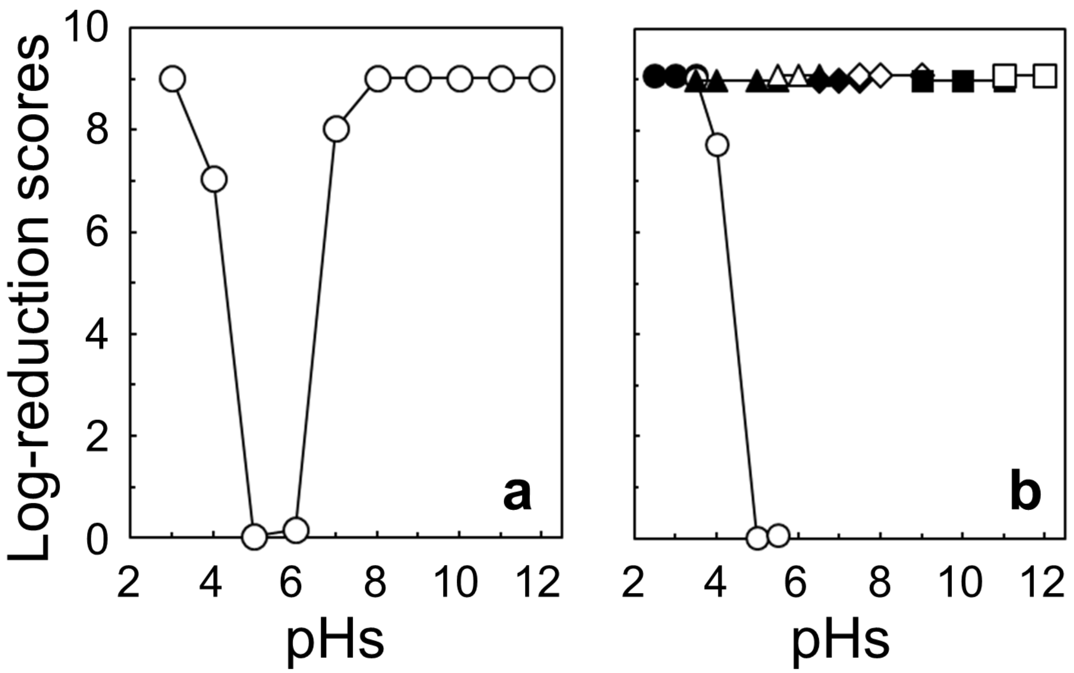

Supplementary Figure 5. pH-response tests of PGA/DEQ coatings.
Panel **a**: Contrary to a general view that PGAIC (*e.g.*, PGA/HDP) coatings are labile following soaking in the universal buffer "Carmody" (○) composed of borate, citrate, and phosphate[1,2], PGA/DEQ exhibited increased resistance to the same buffer (pH 3–12), except in the range pH 5–6, in which citrate molecules are mainly (theoretically) transformed into the *di*-sodium form. **b** Further experiments using a combination set (each 0.1 M) comprising several buffers [glycine-HCl (●; pH 2.5–3.5); Na citrate (○; 3.5–5.5); Na acetate (▲; 3.5–5.5), MES-NaOH (△; 5.5–6.5); MOPS-NAOH (◆; 6.5–7.5); HEPES-NaOH (◇; 7.5–9.0); glycine-KOH (■; 9.0–11.0); and Na_2HPO_4-NaOH (□; 11.0–12.0)][3] also suggested that citrate had a peculiar effect on the extremely stable PGAIC antimicrobials. Retained antimicrobial performance was assessed by comparing log-reduction scores ($n = 5$) in the presence of PGA/DEQ-coated disks after soaking with the indicated buffers. In the present experiment, all the estimated standard deviation scores were actually $<5\%$ (0 to a maximum of 0.26). These imply that the removability of PGA/DEQ coatings, which are extraordinary durable over various pH ranges, can be controlled by adjusting the proportion of certain buffer components.

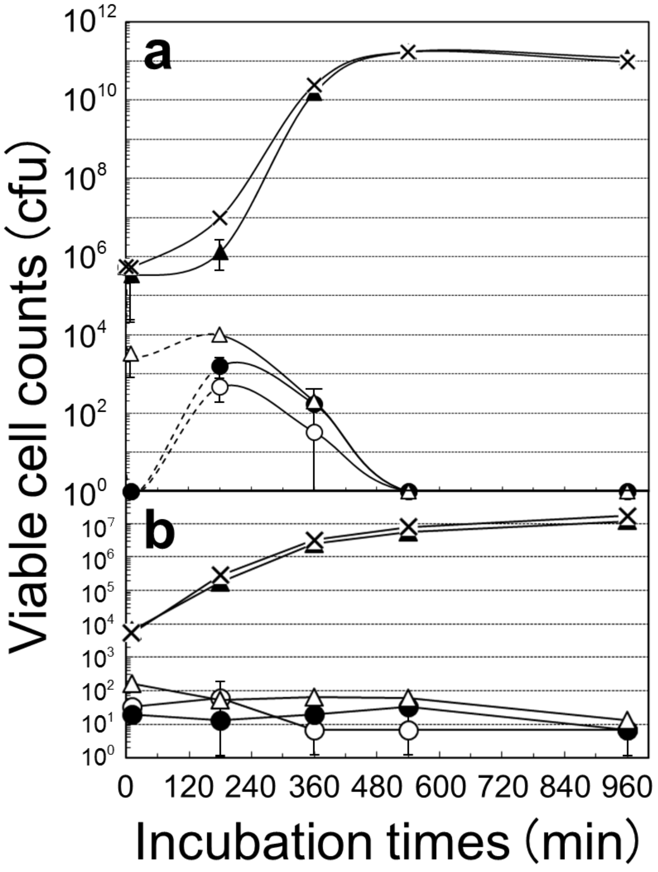

Supplementary Figure 6. Potent antimicrobial performance of PGAIC coatings.

E. coli cells (~ 5.5×10^5 CFU) were inoculated into LB media (5 mL), each carrying a disk (12 mm *dia.*; 0.35 mm *thick*) from the non-coated (*crosses*), and PGA/DEQ- (*circles*) and PGA/HDP-coated (*triangles*) sheets before (*open symbols*) and after (*closed symbols*) the severe treatment using EtOH (*see* Fig. 5). (**a**) Viable cell counts in the liquid culture media ($n = 3$), indicating the expression of a fast elimination (or killing) mechanism (within 10 min), followed by sustainable antimicrobial performance (after 180 min). Particularly, it is noteworthy to be significant in the durable (*e.g.*, extraordinary water-resistant) PGA/DEQ coatings from the viewpoint of improved contact-killing surfaces[4]. (**b**) Counts of viable cells adhered in the disk samples after cultivation ($n = 3$). The moisture of samples was gently drained, and their weights were calculated to be 28 mg averagely ($n = 15$), the scores of which were virtually constant regardless of the incubation times, presumably owing to the size stability of HIYEX non-woven plastic cloth. Each drained sample was then soaked into 1 mL of 100 mM citrate *di*-salts at 25°C for 10 min, and the resulting suspensions were subjected to the counting experiment of viable cells (*see* the Method section). On the PGA/DEQ-coated disks, the viable cells (*though* their numbers are surely not large) were counted even under the circumstances where *E. coli* cells have disappeared from the liquid media (*e.g.*, after the 540-min incubation), providing insight into a functional surface actively involved in bacteria elimination.

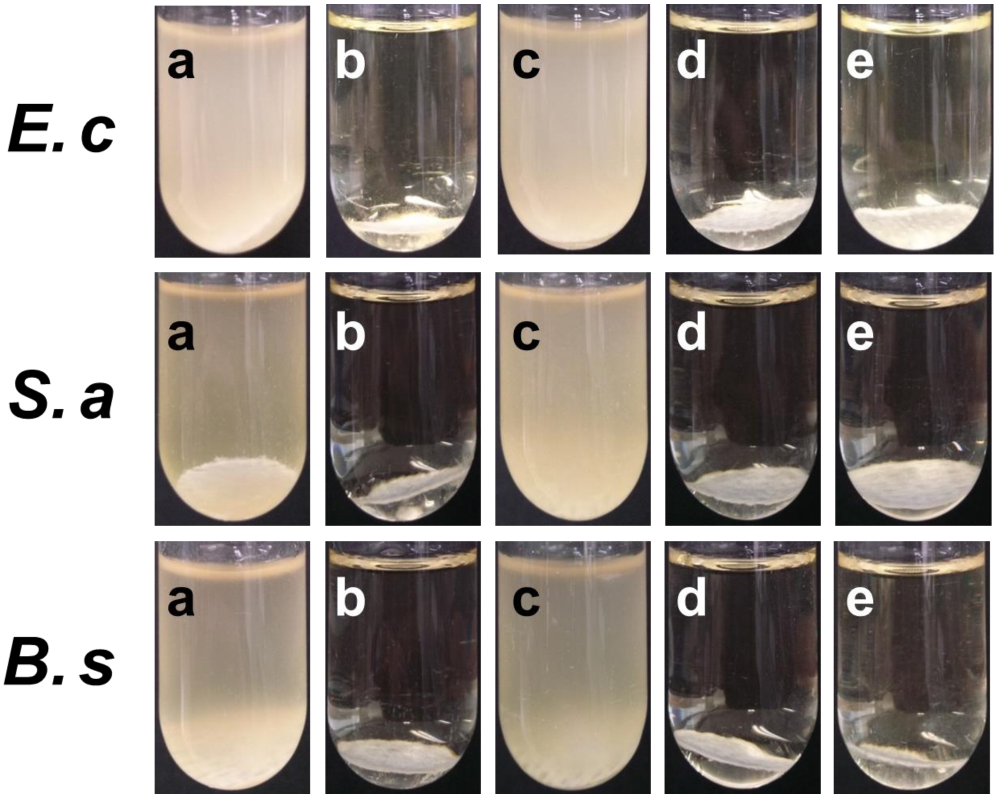

Supplementary Figure 7. Sustainable antimicrobial performance of PGAIC coatings.

Abbreviations: *E.c*, *Escherichia coli*; *S.a*, *Staphylococcus aureus*; *B.s*, *Bacillus subtilis*. Cells of microorganisms ($\sim 5.5 \times 10^5$ CFU) were inoculated into LB media (5 mL), each carrying a disk (12 mm *dia.*) from the non-coated (**a**), PGA/HDP-coated (**b**, before; **c**, after the EtOH soaking), and PGA/DEQ-coated (**d**, before; **e**, after the EtOH soaking) (*see* Fig. 5), and then cultured at 37°C for 5 days. The cultures of images **a** and **c** actually reached to their stationary phase after 24-h incubation, whereas the use of PGA/DEQ coatings (images **d** and **e**) brought about the long-term suppression against cell growth of Gram-positive bacteria (*e.g.*, *S.a* and *B.s*) in addition to Gram-negative bacteria (*e.g.*, *E.c*).

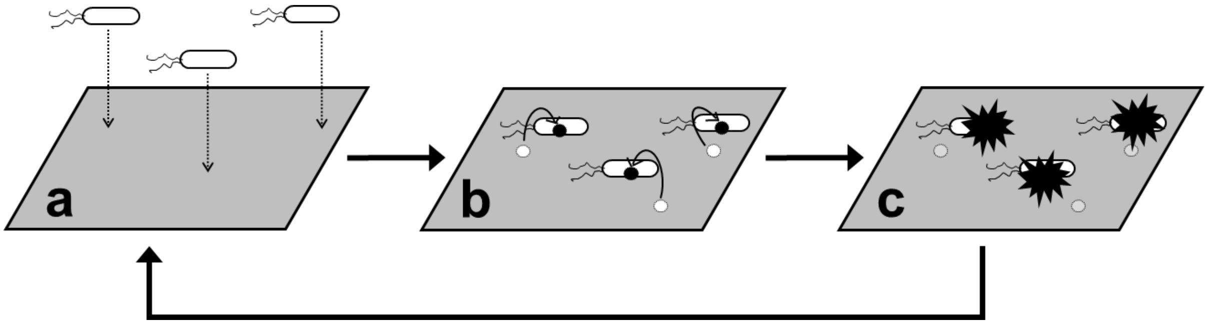

Supplementary Figure 8. Schematic representing a possible novel microbicidal mechanism called “Capture–Killing”.

Steps (**a**), access and capture of microbial cells (or infectious particles); (**b**), highly reliable attack on the captured targets by released drugs; and (**c**), chemical disruption of the captured targets.

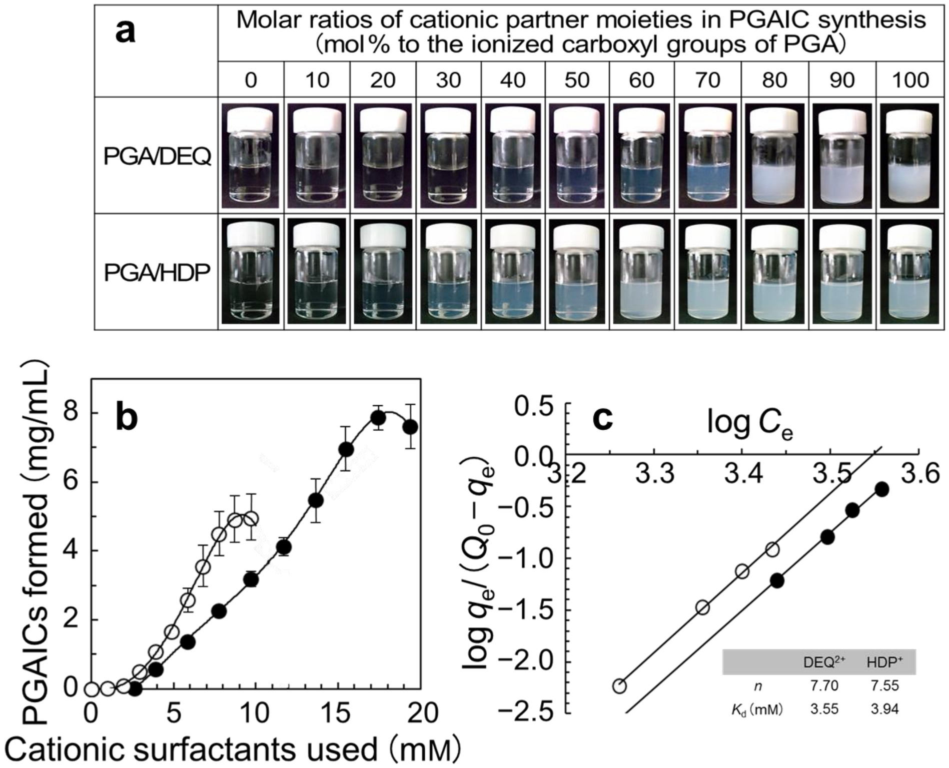

Supplementary Figure 9. Kinetics of PGAIC formation.

The initial concentration (mg/mL) of PGA is 2.5 ± 0.1, indicating the presence of carboxyl residues at ~20 mM. Panel **a**: the dose-dependency of PGAIC (*i.e.* PGA/DEQ and PGA/HDP) formation against the QA-type surfactants used (*i.e.*, DEQ^{2+} and HDP^{+}). The increase in turbidity of the reaction mixtures implied the accumulation of water-insoluble PGAICs. **b** Sigmoid-fitting (*thus* non-hyperbolic) events in the formation of PGAICs (○, PGA/DEQ; ●, PGA/HDP). In the kinetic analysis, the non-ideal competitive adsorption (NICA) model[5–7] (or the Hill equation in enzymology) prefers to the Langmuir model (or the Michaels–Menten equation). **c** Cooperative PGAIC formation was first demonstrated and then kinetically characterized using the NICA model. The cooperativity (n)/affinity (K_d, mM) scores of PGA for DEQ^{2+} and HDP^{+} can be found in the table-type inset. Interestingly, the composition analysis using NMR proved that the carboxyl groups of all the PGAICs in **a** were constantly and completely transformed with QA moieties, presumably owing to their (potent) cooperative bindings.

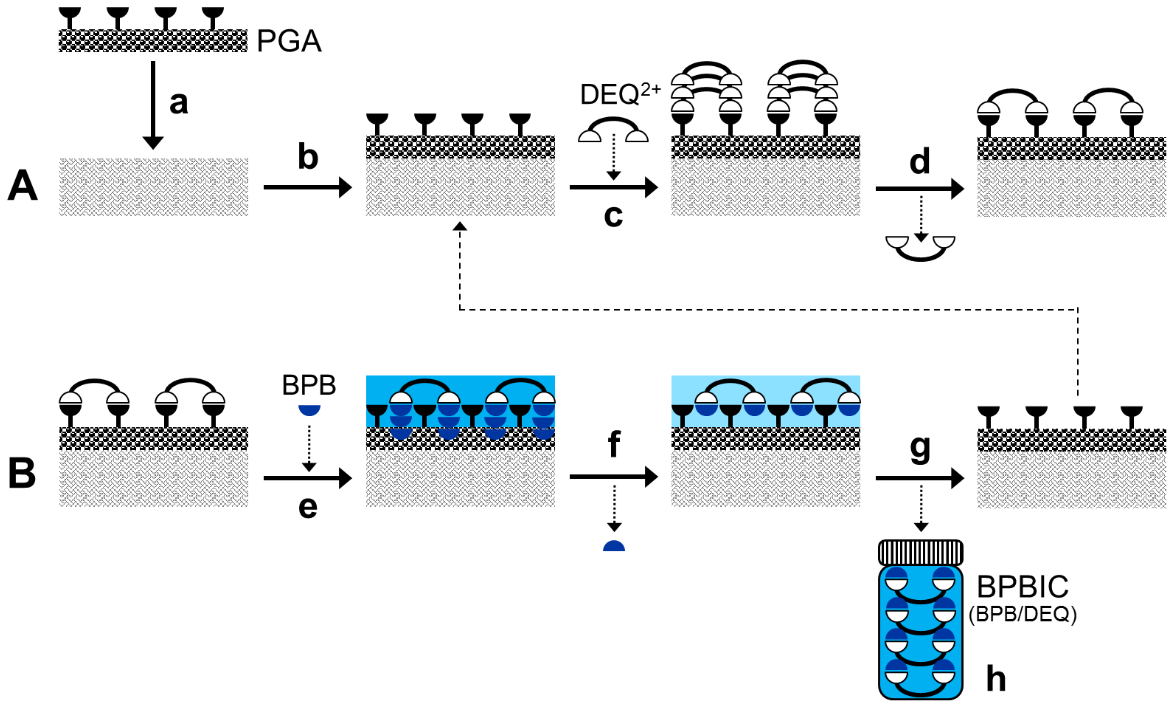

Supplementary Figure 10. Schematic diagrams of (A) the *onsite* synthesis of the PGA/DEQ coatings and (B) their quantitative colorimetric assay.
Steps (**a**), first coating of a PGA solution on the surfaces of base materials; (**b**), surface functionalization *via* the spontaneous coating of PGA as a widely applicable adhesive[8]; (**c**), second coating with a DEQ^{2+} solution on the PGA-mounting surfaces to briefly form PGA/DEQ *onsite*; (**d**), 30-min soaking in methanol (1 mL/disk; repeated a total of three times per treatment process) with gently shaking to wash out excess (unbound) DEQ^{2+} and to leave only durable PGA/DEQ coatings *onsite*; (**e**), 10-min immersion of PGA/DEQ-coated materials in a BPB concentration (1 mL/disk) to form BPB/DEQs (*see* Supplementary Fig. 11, panel **a**); (**f**), 5-min soaking in water (5 mL/disk; repeated five times) to remove unbound BPB anions and remain water-insoluble BPBICs; (**g**), 24-h soaking in methanol (1 mL/disk) to extract BPBIC molecules from the dried surfaces of the resulting disks and ultimately determine the amount of PGAICs thereby immobilized as PGA/DEQ coatings; and (**h**), quantitative analysis of BPBICs (*see* Supplementary Fig. 12).

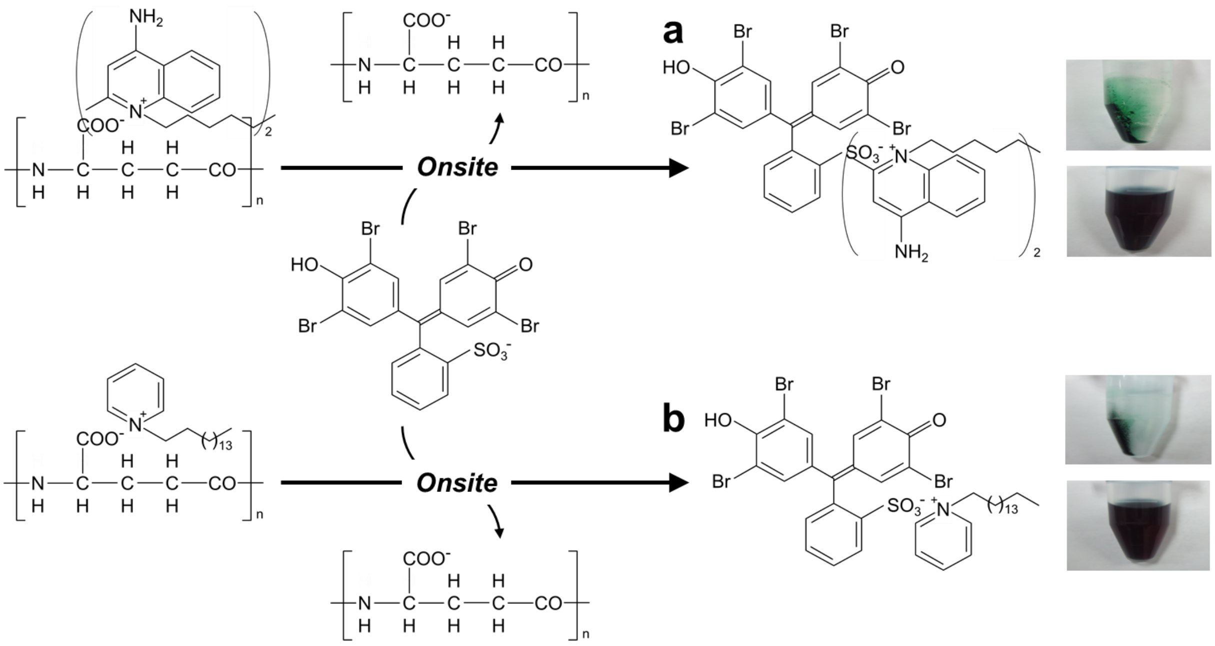

Supplementary Figure 11. Formation of water-insoluble complexes (BPBICs) comprising BPB anions and cationic surfactants on PGAIC-coated surfaces. Predicted structures (**a**), a DEQ-bound form (*namely* BPB/DEQ); and (**b**), an HDP-bound form (BPB/HDP). The inset images depict the solvation of each BPBIC in water (*top*) and methanol (*bottom*).

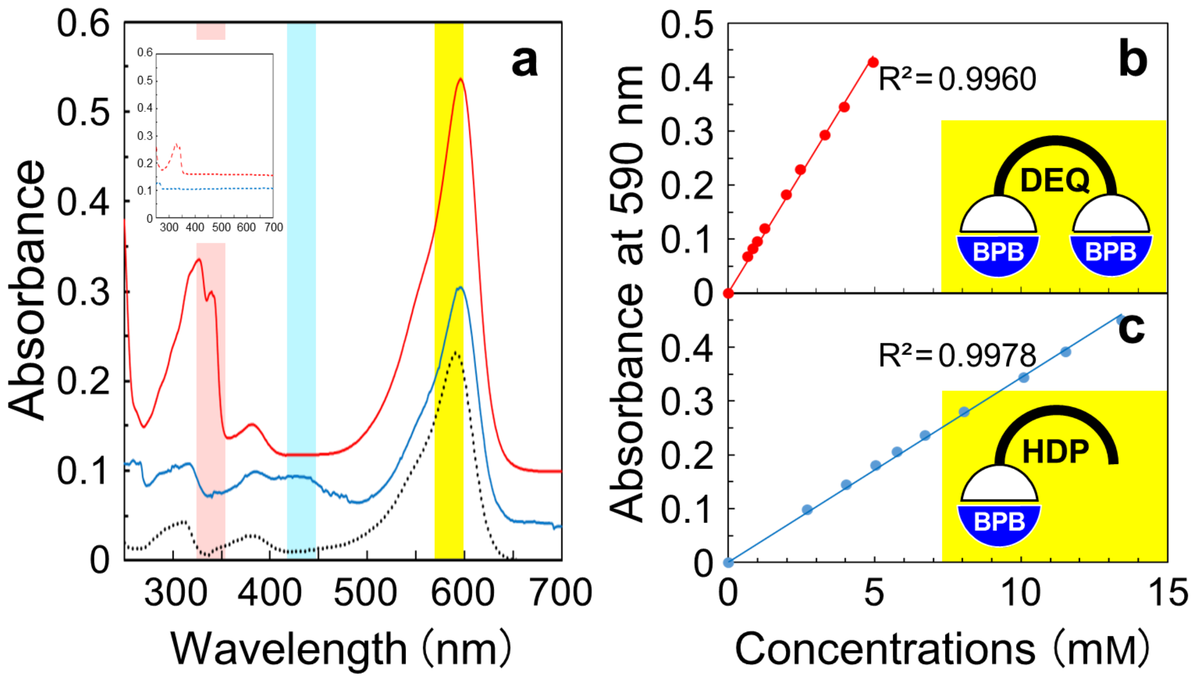

Supplementary Figure 12. Spectrophotometry of BPBICs.

Left panel, (**a**) absorption spectra in methanol of BPB/DEQ (*solid line*, red), BPB/HDP (blue), and free BPB (*dotted line*, black); the inset depicts free DEQ (red) and HDP (blue). The specific absorption of BPB/DEQ is at 330–350 nm (rose-pink zone) and that of BPB/HDP is at 410–450 nm (light-blue zone); however, the maximum absorption wavelength is commonly around 590 nm (yellow zone). *Right* panels, the calibration curves of (**b**) BPB/DEQ and (**c**) BPB/HDP solutions. The insets illustrate the BPB-binding models of BPBIC; hence, a DEQ molecule (*top*) can capture twice the amount of dye as a HDP molecule (*bottom*).

Microorganisms	*Found* concentrations (ppm) [a]	
	DEQ^{2+}	HDP^{+}
Staphylococcus aureus	4	2
Escherichia coli	32	64
Pseudomonas aerginosa	100	>500
Candida albicans	8	25
Aspergillus niger	16	300

Supplementary Table 1. Minimal inhibition concentrations (MICs) of DEQ^{2+} and HDP^{+}. [a]The values were determined according to the guidelines provided by the Clinical and Laboratory Standards Institute (formerly known as the National Committee for Clinical Laboratory Standards)[9].

Supplementary references

1. Carmody, W.R. An easily prepared wide range buffer series. *J. Chem. Edu.* **38**, 559–560 (1961).

2. Ashiuchi, M. & Misono, H. Biochemical evidence that *Escherichia coli hyi* (orf b0508, *gip*) gene. *Biochim. Biophys. Acta* **1435**, 153–159 (1999).

3. Wakamatsu, T., Higashi, C., Ohmori, T., Doi, K. & Ohshima, T. Biochemical characterization of two glutamate dehydrogenases with different cofactor specificities from a hyperthermophilic archaeon *Pyrobaculum calidifontis*. *Extremophiles* **17**, 379–389 (2013).

4. Ashiuchi, M. *et al.* Development of antimicrobial thermoplastic material from archaeal poly-γ-L-glutamate and its nanofabrication. *ACS Appl. Mater. Interfaces* **5**, 1619–1624 (2013).

5. Hakumai, Y., Oike, S., Shibata, Y. & Ashiuchi, M. Cooperative adsorption of critical metal ions using archaeal poly-γ-glutamate. *Biometals* **29**, 527–534 (2016).

6. Koopal, L.K., van Riemsdijk, W.H., de Wit, J.C.M. & Benedetti, M.F. Analytical isotherm equation for multicomponent adsorption to heterogeneous surfaces. *J. Colloid Interface Sci.* **166**, 51–60 (1994).

7. Koopal, L.K., van Riemsdijk, W.H. & Kinniburgh, D.G. Humic matter and contaminants. General aspects and modelling ion binding. *Pure Appl. Chem.* **73**, 2005–2016 (2001).

8. Ashiuchi, M. *et al.* Poly-γ-glutamate-based materials for multiple infection prophylaxis possessing versatile coating performance. *Int. J. Mol. Sci.* **16**, 24588–24599 (2015).

9. Japanese Industrial Standards L 1902. in *Testing for antibacterial activity and efficacy on textile products: English Ed*. 16–19 (Association of Japanese Standards, 2009).
